# Phyto-derived interferons: a promising frontier in antiviral therapy development

**DOI:** 10.17179/excli2024-7998

**Published:** 2025-02-24

**Authors:** Baskar Venkidasamy, Ashok Kumar Balaraman, Muthu Thiruvengadam

**Affiliations:** 1 Department of Oral and Maxillofacial Surgery, Saveetha Dental College and Hospitals, Saveetha Institute of Medical and Technical Sciences, Saveetha University, Chennai, 600077, India; 2 Research and Enterprise, University of Cyberjaya, Persiaran Bestari, Cyber 11, 63000 Cyberjaya, Selangor, Malaysia; 3 Department of Applied Bioscience, College of Life and Environmental Science, Konkuk University, Seoul, Republic of Korea

## ⁯

Interferons (IFNs) are a class of signaling proteins released by host cells in response to pathogens such as bacteria, viruses, and cancer cells, and are central to the host's immune response. IFNs not only inhibit viral replication but also activate immune cells and upregulate MHC (Major histocompatibility complex) molecules, thereby enhancing the body's defense mechanisms against infections and cancer (Abdolvahab et al., 2020[1]). Classified as type I (mainly antiviral IFNs like IFN-α and IFN-β), type II (IFN-γ with roles in inflammatory response), and type III (IFN-λ), these cytokines share a common structure and are characterized by their potent immune-modulatory effects (Cao et al., 2022[5]). Conventional IFN production typically relies on animal cell cultures, which often present high production costs, scalability issues, and the potential for contamination. Plant-based platforms are emerging as viable alternatives, owing to their advantages in terms of cost, scalability, and minimal risk of human pathogen contamination (Takeyama et al., 2015[10]). 

Plants, such as tobacco (*Nicotiana benthamiana*), rice, and radish, have shown potential as biofactories for recombinant IFN production (Supplementary Figure 1). This approach is further supported by advancements in plant biotechnology that allow plants to stably express human IFNs, which are pharmacologically active and suitable for clinical applications. Human interferon-α2a (IFN-α2a) and IFN-α2b have been successfully expressed in radish and tobacco plants, and have exhibited antiviral and anticancer activities. Recent studies have demonstrated that IFN-α2a produced in radishes maintains functional bioactivity and could serve as a low-cost therapeutic option (Kebeish et al., 2022[9]). IFN-α2b has been expressed in the chloroplasts of tobacco plants, and studies have shown its efficacy against various cancers and viral infections (Arlen et al., 2007[3]). *In vivo* assays revealed that plant-derived IFN-α2b enhances immune responses by upregulating *MHC I* expression on immune cells and stimulating NK (Natural Killer) cell production, which is essential for robust antiviral and anticancer defenses. 

One promising technique for increasing IFN yields is the use of plant-based expression vectors. For instance, a modified bamboo mosaic virus (BaMV) vector was applied to *N. benthamiana* to enhance IFNγ production. This vector, pKBΔC_His_, showed the highest expression levels of unmodified IFNγ, and subsequent modifications resulted in biologically active D-form IFNγ in plant leaves (Jiang et al., 2019[8]). A refined BaMV expression system introduced cleavable linkers and optimized processing, resulting in high-purity, tag-free IFNγ. This modified IFNγ demonstrated antiviral efficacy comparable to that of commercial human IFNγ against viruses, such as Sindbis, underlining its therapeutic potential (Jiang et al., 2024[7]). Recent clinical studies have underscored the potential of plant-derived IFNs for therapeutic applications, particularly against respiratory viruses. 

Plant-derived interferons, particularly those bioengineered through molecular farming, represent a promising area for the development of antiviral therapy. Clinical studies have shown that plant-derived interferons effectively combat viral infections by boosting immune responses. For instance, research conducted on hepatitis B and C patients revealed that interferons produced in tobacco plants (via transient expression systems) exhibited antiviral activities comparable to those of traditional recombinant interferons, but with reduced side effects owing to the elimination of mammalian contaminants (Cao et al., 2022[5]). Another study on plant-derived interferons in HIV (Human immunodeficiency virus) management indicated their potential to enhance immune defense mechanisms in patients, although further trials are needed to confirm their clinical efficacy (Boasso, 2009[4]). These advancements suggest that plant-derived interferons could offer an affordable and safer alternative to antiviral therapy, particularly in low-resource settings where traditional biopharmaceuticals are often unaffordable.

Adeosun and Loots (2024[2]) highlighted the role of medicinal plants in enhancing antiviral IFN activity through bioactive metabolites, such as flavonoids, which have synergistic effects with IFNs in inhibiting viral replication. This approach may offer an accessible and sustainable alternative for regions with limited healthcare infrastructure, particularly during a pandemic. Metabolomics has been pivotal in identifying plant-based compounds that boost IFN efficacy, such as terpenoids and flavonoids, which are integral to plant defense and support IFN function. These findings have fueled further research into integrating IFNs with plant-derived compounds to harness their combined effects in enhanced antiviral therapies.

Although it is promising, plant-based IFN production presents several challenges. Key issues include achieving high yields, ensuring stability, and streamlining purification. However, advancements in genetic engineering, such as codon optimization and chloroplast transformation, have helped overcome these obstacles. For example, optimized vectors and expression systems in tobacco plants have achieved sufficient IFN levels for commercial production, up to 20 % of the total soluble protein (Arlen et al., 2007[3]). These improvements pave the way for the large-scale production and clinical translation of plant-derived IFNs. Future research should focus on enhancing plant-based IFN yields and integrating cost-effective purification processes such as simplified protein extraction and stabilizing additives (Castro et al., 2021[6]). The integration of plant-derived IFNs into mainstream medicine depends on the success of clinical trials and regulatory approvals, which are ongoing for certain plant-derived IFNs, particularly those targeting respiratory viruses and certain cancers. The potential of these phytopharmaceuticals to provide high-quality accessible treatments on a global scale may be transformative, particularly in resource-limited settings. 

In summary, IFNs are critical for immune defense against infections and cancers. However, traditional production methods can be costly and pose contamination risks. Plant-based expression offers a promising alternative that combines affordability with scalability and reduces the risk of contamination. The successful production of IFNs such as IFN-α2a and IFNγ in plants such as *N. benthamiana* has demonstrated therapeutic potential against viral infections and cancer, indicating their potential for integration into clinical practice. As technology evolves and regulatory processes streamline, plant-based systems are expected to play an increasingly important role in biopharmaceutical production, meeting the global demand for safe, effective, and accessible treatments. 

## Data availability

All data used are publicly available.

## Supplementary Material

Supplementary information
