# Supplementary material for: Phyto-derived interferons: a promising frontier in antiviral therapy development
Source: EXCLI J. 2025 Feb 24;24:286–8. doi: 10.17179/excli2024-7998 (PMC11956520; doi:10.17179/excli2024-7998)
Supplement: Supplementary information [file EXCLI-24-286-s-001.pdf]

EXCLI Journal 2025;24:286-288 – ISSN 1611-2156
Received: November 04, 2024, accepted: February 18, 2025, published: February 24, 2025

Supplementary information to:

Letter to the editor:

PHYTO-DERIVED INTERFERONS: A PROMISING FRONTIER IN ANTIVIRAL THERAPY DEVELOPMENT

Baskar Venkidasamy[1], Ashok Kumar Balaraman[2], Muthu Thiruvengadam[3,*]

[1] Department of Oral and Maxillofacial Surgery, Saveetha Dental College and Hospitals, Saveetha Institute of Medical and Technical Sciences, Saveetha University, Chennai, 600077, India
[2] Research and Enterprise, University of Cyberjaya, Persiaran Bestari, Cyber 11, 63000 Cyberjaya, Selangor, Malaysia
[3] Department of Applied Bioscience, College of Life and Environmental Science, Konkuk University, Seoul, Republic of Korea

* **Corresponding author:** Dr. Muthu Thiruvengadam, Associate Professor, Department of Applied Bioscience, College of Life and Environmental Science, Konkuk University, Seoul, Republic of Korea. Phone: +82-24500577; E-mail: muthu@konkuk.ac.kr

https://dx.doi.org/10.17179/excli2024-7998

This is an Open Access article distributed under the terms of the Creative Commons Attribution License (http://creativecommons.org/licenses/by/4.0/).

EXCLI Journal 2025;24:286-288 – ISSN 1611-2156
Received: November 04, 2024, accepted: February 18, 2025, published: February 24, 2025

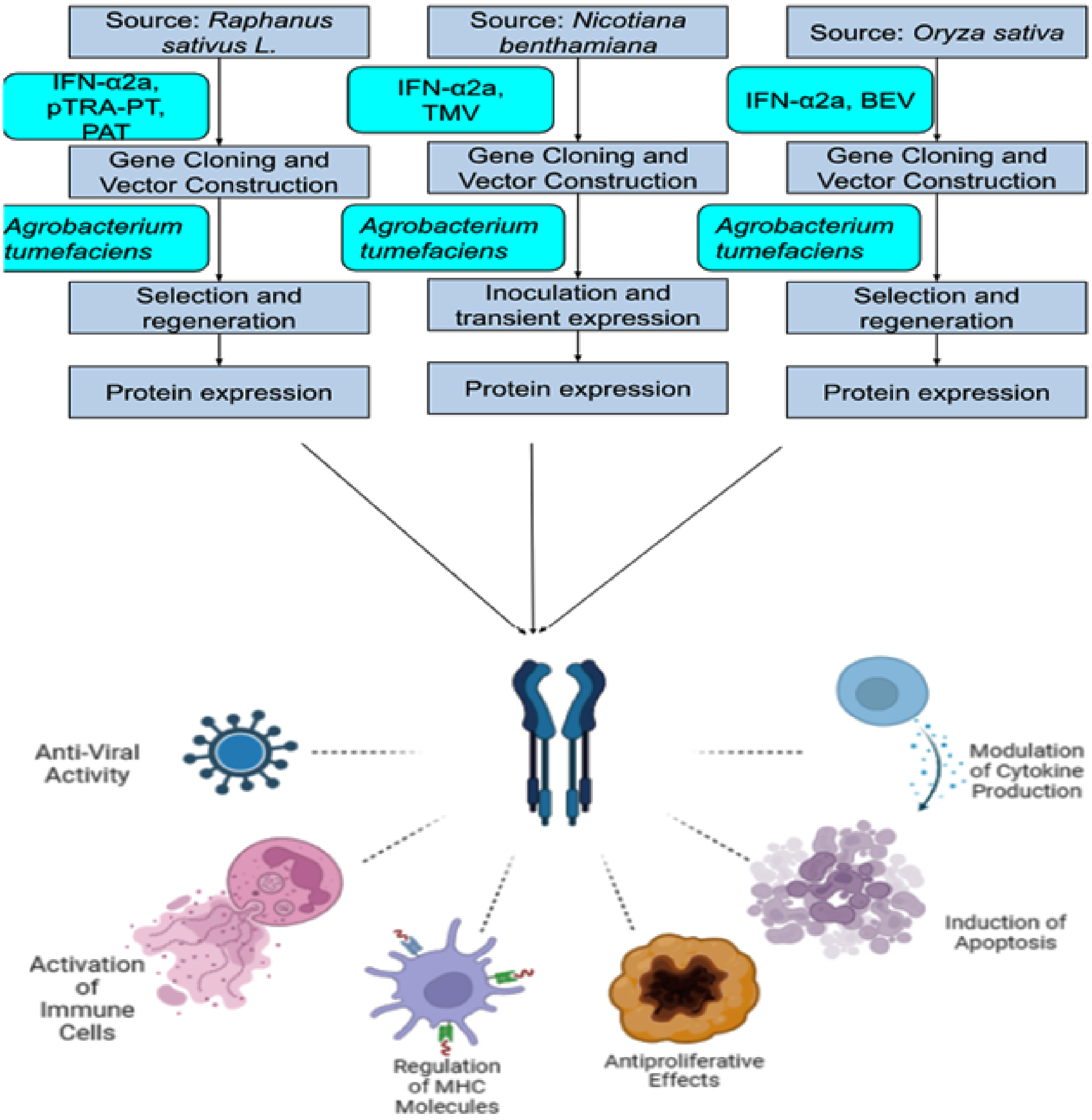

Supplementary Figure 1: Production of plant derived interferons and their applications
